# Root cell wall remodeling during symbiotic microbial colonization

**DOI:** 10.3389/fpls.2026.1765155

**Published:** 2026-02-03

**Authors:** Elizabeth Monroy-Morales, Manoj-Kumar Arthikala, Jesús Montiel

**Affiliations:** 1 Centro de Ciencias Genómicas, Universidad Nacional Autónoma de México (UNAM), Cuernavaca, Mexico; 2 Ciencias Agrogenómicas, Escuela Nacional de Estudios Superiores Unidad León, Universidad Nacional Autónoma de México (UNAM), León, Mexico

**Keywords:** actinorhizal plants, cell wall remodeling enzyme, legume rhizobia symbiosis, mycorrhiza colonization, symbiosis, plant cell wall

## Abstract

Plant roots are usually ground organs that perform essential roles, mostly associated with the anchoring of plants to the soil and absorption of nutrients and water. However, they are also exposed to a wide variety of microorganisms and may develop various symbiotic relationships, such as mutualism, which benefits both organisms. For instance, arbuscular mycorrhizal symbiosis is likely the oldest and most widespread mutualistic association, that occurs between plants and fungi. Another relevant example is the root nodule symbiosis, established between nitrogen-fixing bacteria and nodulating legumes, actinorhizal plants and *Parasponia* species. In both cases, microbial colonization of plant roots culminates in the formation of specialized symbiotic structures. In this regard, microbial infection is a critical step for the mutualistic relationship, where altering the cell wall biomechanics is necessary to facilitate microbial entry, which can be modulated by various cell wall protein families. This review examines the current knowledge on cell wall modifications occurring in plants roots during the symbiotic entry of microorganisms, focusing on the role of cell wall-remodeling proteins involved in these processes.

## 1 Introduction

Plant cell wall is a dynamic structure that plays several essential roles for cell functioning, including support, protection, and regulation of interactions with the environment. It surrounds the plasma membrane and forms a rigid extracellular matrix that maintains cell shape, provides mechanical stability, and acts as the first barrier against internal and external damage (Alonso Baez and Bacete, 2023; Zhang et al., 2025). Structurally, the cell wall is composed of a complex network of polysaccharides, cellulose microfibrils, hemicelluloses, pectins, and structural proteins. Cellulose is synthesized at the plasma membrane by cellulose synthase complexes guided by cortical microtubules, whereas hemicelluloses and pectins are produced in the Golgi apparatus before being secreted into the apoplast (Alonso Baez and Bacete, 2023). This composite architecture provides both rigidity and flexibility, enabling controlled expansion, morphogenesis, and intercellular communication (Zhang et al., 2025).

Besides the relevant functions outlined before, the cell wall also represents an initial point of contact between plant roots and their surrounding microorganisms, playing a key role in mediating symbiotic interactions (Dora et al., 2022). During mutualistic symbioses, including legume-rhizobia root nodule symbiosis (RNS), actinorhizal symbioses and arbuscular mycorrhiza (AM), this barrier must be locally modified to permit microbial entry. Successful colonization requires dynamic modifications of the cell wall, including transient softening, enzymatic loosening, and controlled degradation or restructuring of polysaccharides that allow microbial partners to cross the epidermal and cortical cell wall without compromising host cell viability. Such active cell wall remodeling contributes to creating a transient and highly specialized microenvironment in the apoplast, which supports the initiation and progression of infection structures (Balestrini and Bonfante, 2014; Rich et al., 2014; de Carvalho-Niebel et al., 2024).

In RNS, legumes accommodate nitrogen-fixing rhizobia through intracellular infection pathways involving infection chambers, infection threads, or, in some species, intercellular entry routes (Brewin, 2004; Zhang and Ott, 2024). Actinorhizal plants, by contrast, establish symbioses with filamentous actinobacteria of the genus *Frankia*, typically through intercellular hyphal ingress or infection thread-like structures (Pawlowski and Demchenko, 2012; Fournier et al., 2018). Despite having evolved independently, both types of nitrogen-fixing symbioses rely on precise modulation of the root cell wall to form plant–microbe interfaces where nutrient exchange and nitrogen fixation occur.

Root cell wall remodeling is therefore a central determinant of successful mutualistic colonization. These modifications are triggered by microbial molecular signals and orchestrated by an array of plant-derived components, including cell wall-modifying enzymes (pectin methylesterases, polygalacturonases, pectate lyases, cellulases), structural proteins (extensins, expansins), membrane-bound receptors, transcriptional regulators, and symbiosis-specific genes (Fournier et al., 2008; Balestrini and Bonfante, 2014; de Carvalho-Niebel et al., 2024).

This minireview provides an integrative overview of key cell wall-remodeling proteins that regulate the biomechanics of the plant host cell wall specifically during symbiotic entry. We focus on early infection stages that enable microbial penetration of root tissues in legume-rhizobia symbiosis (including intracellular, intercellular, and crack-entry modes of infection), *Frankia*-actinorhizal symbioses, and arbuscular mycorrhizal colonization. While cell wall dynamics are also relevant beyond the initial phases of symbiosis (Berry et al., 2002; Tsyganova et al., 2009; Li et al., 2015; Su et al., 2023; Gao et al., 2025; Zhao et al., 2025), this review specifically highlights early remodeling events associated with microbial entry.

## 2 Colonization by nitrogen-fixing bacteria

The root nodule symbiosis (RNS) is a mutualistic association, typically observed between soil bacteria known as rhizobia and legumes, as well as the non-legume species *Parasponia* (Behm et al., 2014; Sprent et al., 2017). However, it also occurs in actinorhizal plants, a taxonomically diverse group that engages with actinobacteria *Frankia* spp (Pawlowski and Bisseling, 1996). During rhizobial colonization, the plant cell wall undergoes marked changes in the composition and abundance of structural components, which are required to accommodate bacterial entry and the formation of infection-related structures. These changes are mediated by specific wall-associated proteins that locally remodel host tissues to allow symbiont entry (Murray, 2011; Gao et al., 2024). Root colonization by bacteria is an essential step in the symbiotic process and, depending on the plant host, can occur via two different modalities: intracellular and intercellular (Svistoonoff et al., 2014; Quilbe et al., 2022).

### 2.1 Intracellular infection

**Figure 1:**
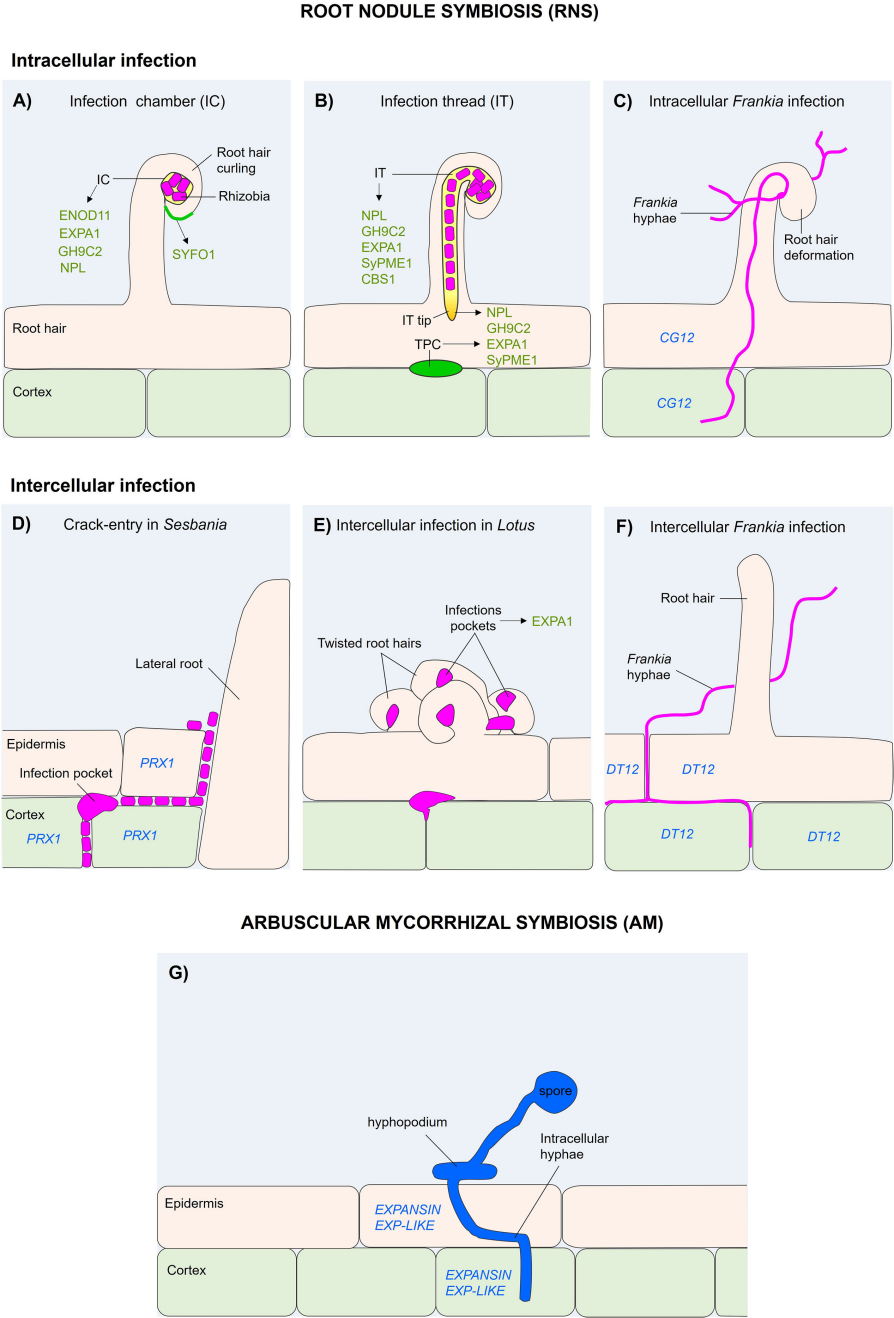
Comparative overview of intracellular and intercellular infection modes and associated cell wall-related gene and protein activity during root nodule symbioses. **(A–C)** Intracellular infection during RNS. **(A)** Intracellular infection via infection chamber (IC) formation in legumes. Compatible rhizobia induce root hair (RH) curling and the formation of an IC, a specialized apoplastic compartment enriched in cell wall-associated proteins involved in early infection, including ENOD11, EXPA1, GH9C2 and NPL, detected under their native promoters. SYFO1 localizes to the root hair tip and curled hairs. *SyPME1 is also detected at the IC under constitutive expression. **(B)** Infection thread (IT) initiation and progression following IC formation during intracellular rhizobial infection in legumes. The IT elongates through root epidermal and cortical cells via polarized growth, involving cell wall remodeling proteins such as NPL, GH9C2, EXPA1, SyPME1 and CBS1. CBS1 localizes along the IT when expressed under the rhizobia-inducible *ENOD12* promoter. Transcellular passage clefts (TPCs) mark sites of IT progression between adjacent cells. **(C)** Intracellular *Frankia* infection via root hair deformation in actinorhizal hosts such as *C*. *glauca*. *Frankia* hyphae enter through deformed root hairs and progress intracellularly toward the cortex. The subtilisin-like serine protease gene *CG12* is expressed in infected root hairs and associated cortical cells during intracellular colonization. **(D–F)** Intercellular infection during RNS. **(D)** Crack-entry infection by *A.caulinodans* in *S. rostrata*. Bacteria invade through epidermal fissures at lateral root bases, forming infection pockets. The class III peroxidase gene *PRX1* is expressed in epidermal and cortical cells surrounding infection pockets formed at lateral root bases. **(E)** Intercellular infection by *Agrobacterium pusense* IRBG74 in *L. japonicus*. Bacteria enter through intercellular infection pockets (IPs) formed between twisted root hairs. The α-expansin EXPA1 localizes to the cell wall matrix of these IPs and is, to date, the only cell wall-related protein reported for this infection mode. **(F)** Intercellular *Frankia* infection in actinorhizal hosts such as *D*. *trinervis*, occurring in the absence of root hair deformation. The subtilisin-like serine protease gene *DT12* is specifically expressed at sites of intercellular colonization along epidermal and cortical infection routes. **(G)** Arbuscular mycorrhizal (AM) symbiosis. Formation of a hyphopodium and intracellular hyphal penetration by arbuscular mycorrhizal fungi, associated with the expression of *EXPANSIN* and *EXPANSIN-LIKE* genes in epidermal and cortical cells. Proteins are indicated by non-italicized labels, whereas gene names are shown in italics. Abbreviations: RNS, root nodule symbiosis; RH, root hair; IC, infection chamber; IT, infection thread; TPC, transcellular passage cleft; IP, infection pocket. Color code: green, protein localization; blue, gene expression.

In legumes, compatible rhizobia attach to root hair tips, inducing their deformation and curling, which leads to the formation of an infection chamber (IC) that encloses the bacteria (**Figure 1A**). From the IC, an inward-growing tubular structure known as the infection thread (IT) develops through coordinated invagination of the host cell wall and plasma membrane (**Figure 1B**), allowing bacterial progression toward inner root tissues (de Carvalho-Niebel et al., 2024; Gao et al., 2024). The progression of IT across cells occurs via a modified apoplastic site known as transcellular passage cleft (TPC) (Zhang and Ott, 2024). Interestingly, although the IC and IT originate from the root hair cell wall, their cell wall composition is apparently different in these structures. In *Medicago truncatula* root hairs infected by *Sinorhizobium meliloti*, the cellulose-binding fluorescent dye calcofluor white, notably accumulates in the cell walls of the developing IC and IT, while accumulating to a lesser extent in the root hair cell wall (Sinharoy et al., 2016).

Recent transcriptome analyses using single-cell RNA sequencing in *M. truncatula* and *Lotus japonicus* revealed that a wide array of genes encoding cell wall remodeling proteins exhibit differential expression during intracellular rhizobial infection (Cervantes-Perez et al., 2023; Frank et al., 2023; Pereira et al., 2024). This evidence is further supported by the detection of the encoded proteins at key structures and events of this process. In *M. truncatula*, symbiotic formin 1 (SYFO1), a plasma membrane protein with an extracellular domain, is enriched at the root hair tips and curled hairs upon rhizobial inoculation. The typical root hair responses, IC formation and actin filaments rearrangements are severely compromised in *syfo1* mutants during *S. meliloti* colonization, confirming the key role of SYFO1 in the interplay between cell wall, plasma membrane and actin cytoskeleton at the initial steps of rhizobial colonization (Liang et al., 2021).

In a subsequent stage, a distinct set of apoplastic proteins accumulates at the IC matrix (**Figure 1A**). Among these, early nodulin 11 (ENOD11), a repetitive proline-rich protein, is enriched at the IC cell wall and has become a hallmark marker of early infection stages (Fournier et al., 2015). In parallel, several enzymes with cell wall-loosening activity converge at this site, including the α-expansin EXPA1, the endoglucanase Glycoside Hydrolase 9C2 (GH9C2) and the nodulation pectate lyase (NPL). Loss-of-function mutants in these genes display severe defects in IT initiation and progression, indicating that multiple apoplastic proteins with cell wall-loosening activity converge at the IC matrix to promote IT emergence (Xie et al., 2012; Su et al., 2023; Montiel et al., 2025; Zhao et al., 2025). In addition, the symbiosis-specific pectin methylesterase SyPME1 has been detected in root hairs surrounding the IC under constitutive expression conditions, and functional inhibition assays indicate that PME activity is required at the IC to enable infection thread emergence (Su et al., 2023).

In the infective structures of subsequent stages, such as the growing ITs and the TPC the abundance and composition of the aforementioned cell wall proteins slightly differ (**Figure 1B**). For instance, the *M. truncatula* cystathionine-β-synthase-like domain-containing protein (*Mt*CBS1) accumulates within the IT matrix, where it might contribute to cell wall maturation (Sinharoy et al., 2016). In contrast, EXPA1 and SYFO1 are barely found along the ITs (Liang et al., 2021). However, EXPA1 and NPL are strongly enriched at TPCs, indicating a specialized requirement for cell wall loosening during transcellular IT passage (Su et al., 2023; Montiel et al., 2025). Regarding SyPME1 and GH9C2, these proteins are detected along the ITs throughout the intracellular infection process, including the TPC (Su et al., 2023; Zhao et al., 2025). Overall, these studies demonstrate the essential role played by these cell wall remodeling proteins at different steps of the intracellular colonization in legumes. Additional cell wall-related proteins, including class III peroxidases and cellulose synthase-like D1 (CSLD1), have also been implicated; however, their precise spatial distribution and functional contributions remain to be elucidated (Ramu et al., 2002; Breakspear et al., 2014; Karas et al., 2021).

Intracellular colonization is not restricted to legumes. In actinorhizal plants, compatible *Frankia* strains can also infect through root hairs (**Figure 1C**), inducing their deformation and initiating infection in multilobed hairs at lobe junctions or shared cell walls (Berry et al., 1986; Newcomb and Wood, 1987). This process leads to the encapsulation of the *Frankia* hypha by a plant cell wall derived structure where subsequently the IT is formed (Pawlowski and Demchenko, 2012). Although certain studies suggest that *Frankia* might contribute to IT progression with the secretion of cell wall degrading enzymes, the evidence collected is not conclusive since the secretome of different *Frankia* symbionts reveal low plant cell wall degrading capacity (Muller et al., 1991; Benoist et al., 1992; Mastronunzio et al., 2008). On the plant side, the transcriptome response of *Casuarina glauca* and *Alnus glutinosa* roots infected by *Frankia* reveals that genes encoding cell wall remodeling proteins are remarkably upregulated (Hocher et al., 2011). In *C. glauca*, the *CG12* gene, encoding an apoplastic subtilisin-like serine protease is highly expressed in infected root hairs, where it may contribute to cell wall loosening during intracellular colonization (**Figure 1C**) (Svistoonoff et al., 2003).

Expansins contribute to host cell wall remodeling during RNS. In *L. japonicus*, the α-expansin EXPA1 localizes to infection-related structures during both intracellular and intercellular colonization, supporting a direct role in infection progression (Montiel et al., 2025).

### 2.2 Intercellular infection

Different lines of evidence suggest that intercellular invasion of rhizobia is a fundamental and ancient mechanism for symbiotic root colonization in legumes, which is preserved in around 25% of all nodulating genera (Sprent, 2007). This process includes different modalities, such as the crack entry at lateral root bases (LRB) or between the epidermal and root hair cells (Ibañez et al., 2017). Regardless the type of intercellular infection, genes encoding cell wall remodeling proteins are among the most upregulated in roots of different legume species (Chaintreuil et al., 2016; Peng et al., 2017; Gully et al., 2018; Karmakar et al., 2019; Montiel et al., 2021; Raul et al., 2022; Camuel et al., 2024).

Functional evidence for the involvement of specific cell wall remodeling proteins during intercellular colonization remains limited. In *Sesbania rostrata*, intercellular infection occurs via crack entry at lateral root bases (**Figure 1D**), and during this process the class III peroxidase *SrPRX1* is strongly induced in response to *Azorhizobium caulinodans*. *SrPRX1* transcripts accumulate specifically at the infection pocket (IP) and in the surrounding host cells during crack-entry intercellular infection (Den Herder et al., 2007). Similarly, the *L. japonicus* the α-expansin EXPA1 localizes to the cell wall matrix of IPs during intercellular colonization by *Agrobacterium pusense* IRBG74 (**Figure 1E**). Both the intercellular colonization and nodule organogenesis are severely compromised in the *Ljexpa1* mutants, highlighting the relevant role played by EXPA1 in these processes (Montiel et al., 2025). In addition, both *Lj*NPL and *Lj*CBS are likely participating in the early steps of the *Lotus*-IRBG74 symbiosis, since *Ljnpl* and *Ljcbs* mutants exhibit a drastic reduction in nodule formation in response to IRBG74 inoculation (Montiel et al., 2021).

Intercellular infection has also been described in certain actinorhizal plants. In *Discaria trinervis*, filamentous *Frankia* gains access to the root through intercellular junctions between epidermal and cortical cells (**Figure 1F**). During this process, the *DT12* gene, encoding a subtilisin-like serine protease and homologous to the *C. glauca CG12* gene, is specifically expressed at sites of intercellular colonization, providing molecular evidence for host-mediated apoplastic modification during bacterial entry (Fournier et al., 2018). Interestingly, the intercellular spaces colonized by *Frankia* are devoid of SYTO9 staining, suggesting a distinct composition of the cell wall matrix. This observation provides indirect evidence for host apoplastic remodeling during intercellular colonization and is consistent with earlier work in *Ceanothus* nodules showing that the modified apoplastic compartment formed after *Frankia* infection is enriched in pectic polysaccharides (Liu & Berry, 1991). Together, these findings support a role for targeted host cell wall remodeling in facilitating intercellular colonization in actinorhizal symbioses.

Notably, the infection-associated gene *MtENOD11*, which encodes a cell wall protein in *M. truncatula*, is transcriptionally activated during *Frankia* colonization in both *D. trinervis* and *C. glauca*, suggesting conservation of infection-related transcriptional programs across actinorhizal and legume symbioses, independently of the infection route (Fournier et al., 2018).

## 3 Cell wall remodeling during mycorrhizal colonization

Arbuscular mycorrhizal (AM) fungi establish the most widespread endosymbiotic association in plants, forming mutualistic relationships with nearly 80-90% of terrestrial species. Similar to the RNS, AM colonization relies on the intracellular accommodation of a microbial partner and on a host-driven developmental program that enables fungal ingress into cortical cells (Balestrini and Bonfante, 2014). A hallmark of AM symbiosis is the formation of the intracellular arbuscule, a highly branched fungal structure surrounded by a plant-derived interface that enables efficient nutrient exchange (Bonfante and Genre, 2010; Keegstra, 2010). The establishment of arbuscular mycorrhizal (AM) symbiosis in legumes involves extensive restructuring of plant root tissues, particularly the cell wall, to accommodate the fungal partner while maintaining cellular integrity and function. This remodeling process enables the creation of a specialized compartment the symbiotic interface which defines the structural hallmark of biotrophic mycorrhizal associations (Scannerini and Bonfantefasolo, 1983; Balestrini et al., 2005b).

### 3.1 The symbiotic interface and biotrophy

AM colonization begins at the epidermis, where the fungal hyphopodium establishes contact with root cells (**Figure 1**). Beneath this site, the host organizes a prepenetration apparatus (PPA)-a transient cytoskeletal and endoplasmic reticulum-based structure that guides fungal ingress while maintaining separation between plant and fungal cytoplasms (Genre et al., 2008). This event triggers the synthesis of a host-derived membrane, the perifungal membrane, delimiting the interface zone where molecular exchange occurs. Between this membrane and the fungal wall lies the interfacial matrix, a modified apoplastic compartment enriched with cell wall–like materials (Bonfante, 2001). Vesicle trafficking mediated by VAMPs contributes to membrane proliferation and interface formation, reflecting molecular parallels between symbiotic interface construction and plant cell wall biogenesis during cytokinesis (Genre et al., 2011; Ivanov et al., 2012).

### 3.2 Remodeling during fungal entry and interface formation

The AM fungus does not degrade the host wall enzymatically but induces the plant to remodel it locally during penetration. Beneath the hyphopodium, localized wall softening and rearrangement of cellulose microfibrils facilitate fungal entry without compromising cell integrity (Genre et al., 2008). Microscopy studies show that this remodeling extends along the entire PPA trajectory, forming an invaginated membrane continuum around the advancing hypha (Genre et al., 2011). This controlled remodeling contrasts with pathogen-induced degradation, underscoring the cooperative nature of symbiotic accommodation.

Once the hypha is inside the host cell, it becomes surrounded by the perifungal membrane and the interfacial matrix. The latter comprises plant-secreted polysaccharides and glycoproteins such as β-1,4-glucans, homogalacturonans, xyloglucans, hydroxyproline-rich glycoproteins (HRGPs), and arabinogalactan proteins (AGPs) (Bonfante-Fasolo et al., 1991; Balestrini et al., 1996). Immunocytochemical analyses confirm that these components are synthesized *de novo* and delivered via vesicular secretion guided by the PPA (Genre et al., 2013). Structurally, the interfacial matrix differs from the primary cell wall: it is amorphous, heterogeneous in thickness, and dynamically remodeled during arbuscule development. It tends to be thicker around the arbuscule trunk and thinner at fine branches, consistent with localized variations in nutrient exchange (Balestrini and Bonfante, 2005). AM colonization can also increase cortical cell wall thickness, suggesting that the symbiotic interface requires both loosening and reinforcement at different stages. Expansin proteins, detected at both the host wall and the interface, are thought to maintain the flexibility necessary for fungal accommodation (Balestrini et al., 2005a).

### 3.3 Regulation of cell wall remodeling during AM symbiosis

AM colonization triggers strong transcriptional reprogramming of cell wall–related genes in *M. truncatula* and *L. japonicus* (Guether et al., 2009). Genes encoding HRGPs and AGPs are highly expressed in arbusculated cells (van Buuren et al., 1999). The xyloglucan endotransglucosylase/hydrolase MtXHT1 restructures xyloglucan–cellulose linkages to facilitate fungal accommodation (Maldonado-Mendoza et al., 2005). Transcriptomic analyses in *M. truncatula* revealed the early induction of *EXPANSIN* and *EXPANSIN-LIKE* genes during hyphopodium formation and intracellular penetration by *Rhizophagus irregularis*, suggesting a role for cell wall loosening in facilitating fungal entry across epidermal and cortical tissue (**Figure 1**) (Siciliano et al., 2007). The activation of *ENOD11* in arbusculated cells illustrates the convergence of regulatory pathways between rhizobial and mycorrhizal symbioses (Chabaud et al., 2002). Other genes, including endo-β-1,4-glucanases (*LjCEL1*) and cellulose synthases (*LjCESA*), are also strongly upregulated (Guether et al., 2009). Proteomic studies add another layer of complexity, revealing the involvement of subtilases (*Lj*SBTM1, *Lj*SBTM3) and ascorbate oxidase (*Lj*AO1) in proteolytic and oxidative remodeling of wall components (Takeda et al., 2009; Balestrini et al., 2012).

The interplay between plant and fungal wall dynamics maintains cellular compartmentalization while enabling metabolic cooperation (Harrison, 1999). Similar wall-remodeling genes in rhizobial and mycorrhizal symbioses indicate an evolutionary shared toolkit (Parniske, 2008). AM symbiosis also influences environmental interaction: AMF inoculation can remodel root wall biosynthesis and increase Cd fixation in walls, reducing Cd translocation from root to shoot (Gao et al., 2021). Furthermore, AM-defective mutants display altered regulation of wall-related genes, highlighting the contribution of cell wall plasticity to nutrient uptake efficiency (Willmann et al., 2013).

## 4 Summary and outlook

Cell wall remodeling during AM colonization in legumes involves synthesis, loosening, reinforcement, and dynamic reorganization of wall components. The plant reprograms wall-related gene networks, cytoskeletal organization, and secretory pathways, while the fungus modifies its own wall and provides signaling molecules that sustain compatibility. AM symbiosis also contributes to systemic defense priming, including cellulose accumulation, pectinesterase suppression, cuticle remodeling, miRNA-mediated regulation, and even enhanced callose deposition, shown by the fivefold increase observed when *R. irregularis* acts together with *Pseudomonas putida* KT2440 (Pérez-de-Luque et al., 2017; German et al., 2023). Future research combining single-cell transcriptomics and high-resolution imaging will clarify how localized and systemic wall remodeling contribute to symbiotic performance and resilience in legumes.

The collective evidence demonstrates that root colonization by symbiotic microorganisms relies on a broad array of plant cell wall remodeling proteins that selectively loosen or reinforce the wall at infection sites. Interestingly, many of these proteins belong to large multigene families, suggesting that specific isoforms have been recruited by the plant host to regulate the entry of mutualistic microorganisms. However, as highlighted in this minireview, the molecular basis of intercellular infection remains largely unknown, although important progress is emerging from new model systems such as *Aeschynomene- Bradyrhizobium* and *Lotus*-IRBG74 (Montiel et al., 2021; Camuel et al., 2024).

The next step in understanding the relevance of cell wall dynamics during symbiotic colonization is to integrate wall remodeling with other cellular processes, such as plasma membrane reorganization, lipid dynamics, and cytoskeletal architecture, toward a holistic understanding of how plants accommodate their symbiotic partners.
